# Insights into the Solution Structure and Oligomeric State of Fructose-1,6-bisphosphate Aldolase and Pyruvate Kinase from *Nakaseomyces glabratus* by Small-Angle X‑ray Scattering (SAXS) and AlphaFold Prediction

**DOI:** 10.1021/acsomega.6c06099

**Published:** 2026-09-03

**Authors:** Mayra Cuéllar-Cruz, Dritan Siliqi, Abel Moreno

**Affiliations:** † Departamento de Biología, División de Ciencias Naturales y Exactas, Campus Guanajuato, 56078 Universidad de Guanajuato, Noria Alta S/N, Col. Noria Alta, 36050 Guanajuato, Guanajuato, México; ‡ 296400 Istituto di Cristallografia, Consiglio Nazionale delle Ricerche, Via Giovanni Amendola 122/O, 70126 Bari, Italy; § Instituto de Química, Universidad Nacional Autónoma de México, Av. Universidad 3000, Ciudad Universitaria, 04510 Ciudad de México, México

## Abstract

*Nakaseomyces glabratus* is an opportunistic pathogen of humans, causing invasive candidiasis (IC). Among the risk factors that favor IC are various host-specific factors. Although drugs are available to treat candidiasis, their clinical application remains limited. Therefore, it is necessary to identify therapeutic targets that will enable the development of new antifungals. In this regard, our research group has identified enzymes as potential therapeutic targets in *N. glabratus*, including fructose-1,6-bisphosphate aldolase (Fba1) and pyruvate kinase (Pk). Enzyme activity studies on these two enzymes have shown that they are important therapeutic targets against this pathogen. However, their three-dimensional structure has not yet been elucidated, an essential requirement for designating an enzyme as a therapeutic target. To propose Fba1 and Pk of *N. glabratus* as potential therapeutic targets, we investigated the solution structure and oligomeric state of *N. glabratus* Fba1 and Pk for the first time by combining Small-Angle X-ray Scattering (SAXS) with AlphaFold3 modeling. SAXS data were collected on the B21 beamline at Diamond Light Source (Didcot, UK), providing solution-scattering profiles, molecular-weight estimates, and low-resolution molecular envelopes. These data indicate that Pk is monomeric and Fba1 homodimeric in solution were used to evaluate and refine the corresponding AlphaFold3 atomic models by molecular dynamics. These structural findings for Fba1 and Pk from *N. glabratus* open the door to understanding these enzymes as potential therapeutic targets against this pathogen and, at the same time, a basis for future comparative studies.

## Graphical abstract

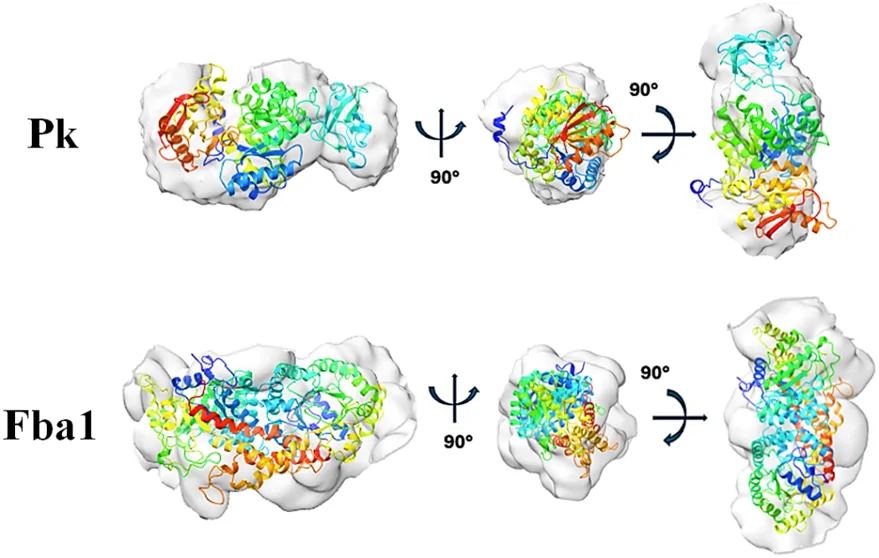

## 1 Introduction

Invasive fungal infections caused by *Candida* species are associated with high mortality among patients hospitalized in intensive care units, tertiary care centers, and general hospitals.^1,2^ Candidemia is the most common clinical presentation of invasive candidiasis (IC), affecting one or more organs in the patient.^1,3^ Risk factors associated with IC include host-related factors, antifungal resistance, length of stay in the intensive care unit, use of broad-spectrum antibiotics, use of central venous catheters, immunosuppressive therapy, and abdominal surgery.^3,4^ However, although drugs for the treatment of candidiasis are currently available, their clinical application is limited. Therefore, it is necessary to identify therapeutic targets that will enable the development of new antifungals. In this regard, our research group has identified enzymes as potential therapeutic targets in *Nakaseomyces glabratus*, including fructose-1,6-bisphosphate aldolase (Fba1) and pyruvate kinase (Pk).^5–7^ This is because both proteins in *Candida albicans* and *Nakaseomyces glabratus* share a low sequence similarity with their counterparts in *Homo sapiens* (20–50% identity).^7^ This large evolutionary distance indicates that they are ideal candidates for therapeutic targets in the design of antifungals, as they would allow attacking the fungus without harming the human host.^7^ Enzyme activity studies targeting these two enzymes have shown that they are important therapeutic targets against this pathogen.^7,8^ However, their three-dimensional structure has not yet been elucidated, which is an essential requirement for identifying an enzyme as a therapeutic target, as it allows for the direct determination of the structure–activity relationship of known active compounds and enables modifications that do not interfere with binding but that favor other parameters such as affinity, selectivity, hydrophobicity, or ionization.^9,10^ With the aim of proposing Fba1 and Pk from *N. glabratus* as potential therapeutic targets, this study set out to determine their three-dimensional structures for the first time using Small-Angle X-ray Scattering (SAXS). This technique is a powerful biophysical method that provides information on the shape, conformation, and assembly state of proteins.^11–13^ To this end, SAXS data for *N. glabratus* Pk and Fba1 were collected on the B21 beamline at the Diamond Light Source (Didcot, UK).^14^ The 3D structures of both enzymes were determined using this technique. The elucidation of the 3D structures of Fba1 and Pk from *N. glabratus* in this study opens the door to understanding these enzymes as potential therapeutic targets against this pathogen and provides insights into the evolution of these proteins in yeast.

## 2 Experimental Section

### 2.1 Strains and Culture Conditions

The strains used in this study were pET19b-FBA-Cg and pET19b-PK-Cg (previously reported by^8^ Medrano-Díaz et al., 2018), which correspond to the *Escherichia coli* (*E. coli*) strain BL21 (DE3), transformed with the pET19b vector containing the open reading frame of the *FBA1* or *PK* gene from *N. glabratus*, Amp^R^. *E. coli* cells were grown in Luria–Bertani (LB) medium at pH 7.0, which contains: biotryptic peptone 10 g/L; NaCl 5 g/L; yeast extract 5 g/L. For solid LB, 2% bacteriological agar was added. In the selection assays, 10 μg/mL kanamycin (Kn) and/or 100 μg/mL ampicillin (Amp) were added to the medium. Liquid cultures were incubated with constant orbital agitation at 250 rpm at 37 °C.

### 2.2 Design and Construction of the Plasmid for the Overexpression of Fba1 and Pk from *N. glabratus*

The design accounted for codon optimization, as codons differ between *E. coli* and *N. glabratus* has been previously reported.^8^ Briefly, the methodology used is described below: the optimized gene sequence for FBA (CgFba1 ID: KTB27082.1) or PK (CDC19 CgPk ID: XP_449865.1) was sent to GenScript (Piscataway, New Jersey, USA) for synthesis, based on the original protein sequence (the *Candida* Genome Database reference sequence for *N. glabratus* CBS138). Restriction sites recognized by the *XhoI* and *Bam*HI enzymes were added to the 5′ and 3′ ends of the synthesized sequences, respectively, which adds a 6-histidine tag (His6X) to the recombinant protein. The optimized gene sequences were received as inserts in the pET19b expression plasmid.^8^

### 2.3 Obtaining the Recombinant Strains

Competent cells were prepared according to the protocol previously described by,^8,15^ using the PureLinkTM Quick Plasmid Miniprep kit (Invitrogen).

### 2.4 Overexpression of Fba1 and Pk

Based on Medrano-Díaz et al.,^8^ the strain was cultured in LB medium with kanamycin, inducing the expression of recombinant proteins with 0.5 mM IPTG at 37 °C until an OD_600 nm_ of 0.5–0.8 was reached. After 6 h, the cells were harvested, washed, and lysed by sonication in lysis buffer; the soluble fraction was separated from the insoluble fraction by centrifugation, and protein induction and purity were analyzed by 12% SDS-PAGE.^15,16^

### 2.5 Purification of the Recombinant Fba1 and Pk Proteins

Protein purification was performed according to the protocol previously described by Cuéllar-Cruz et al.^15^ Briefly, the protein was purified by affinity chromatography using a HisTrap HP column (1 mL) at a constant flow rate of 0.5 mL/min. After equilibrating the column and loading 1 mL of the sample, successive washes were performed with 50 mM Na_2_HPO_4_·7H_2_O, 500 mM NaCl, and 500 mM imidazole buffer. Subsequently, washing was performed using the same equilibration solution, but 5% glycerol was added (50 mM Na_2_HPO_4_·7H_2_O, 500 mM NaCl, 500 mM imidazole, 5% glycerol). Elution was then performed using the elution buffer containing 50 mM Na_2_HPO_4_·7H_2_O, 500 mM NaCl, 500 mM imidazole, 1 mM PMSF, 5% glycerol, pH 8.0, at a flow rate of 0.5 mL/min and a pressure of 0.5 MPa. Protein elution was performed using a continuous gradient, and 1 mL fractions were collected. The purified protein was quantified using a Nanodrop.

### 2.6 Small-Angle X-ray Scattering (SAXS)

Small-angle X-ray scattering (SAXS) data for *N. glabratus* Pk and Fba1 were collected on the B21 beamline at the Diamond Light Source (Didcot, UK).^14^ The experiments were conducted in 20 mM Tris, 100 mM NaCl, pH 8.0, using an EigerX 4 M detector at a sample–detector distance of 3.7 m and a wavelength of 0.95 Å. In-line size-exclusion chromatography (SEC-SAXS) was employed for both constructs. A 40 μL sample, at the concentration 5 and 1 mg/mL for Pk and Fba1 respectively, was injected at a flow rate of 0.075 mL/min onto a GE Superdex 200 Increase 3.2/300 column at 15 °C. Scattering data were collected over 620 successive 3-s frames.

Data processing involved normalization to the transmitted beam intensity, radial averaging, and subtraction of solvent-blank scattering. ScÅtter software (http://www.bioisis.net/) was used to identify and select frames corresponding to sample scattering and the appropriate solvent blank. Subtracted frames were scaled and averaged to produce the final SAXS profile.

The initial analysis of the SAXS-derived parameters for all the SAXS experiments was performed in ATSAS 4.1 package^17^ and are listed in Table 1.

**Table 1:** SAXS Sample Details, Data Collection, Analysis, and 3D Modeling Details

sample	Pk	Fba1
organism	*N. glabratus*	
**Data Collection Parameters**		
synchrotron beamline	B21/Diamond Light Source (Harwell, UK)	
detector (distance m)	EigerX 4M-Detrics (3.7)	
Beam size (mm)	0.05 × 0.05	
energy (keV)	13.10	
*q* range (nm^–1^)	0.045–3.40	
exposure time (s)	3.0	
number of frames	620	
temperature (K)	287	
mode	SEC online/Superdex 200 3.2/300 inc	
**Structural Parameters**		
injection concentration (mg/mL)	5.0	1.0
*q* Interval for Fourier inversion (nm^–1^)	0.14–2.30	0.18–1.40
*R*_g_ [from P(r)] (nm) GNOM total estimate (evaluation)	3.20 ± 0.20 0.79 (Good)	4.21 ± 0.14 0.65 (Reasonable)
*R*_g_ [from Guiner approximation] (nm)	3.20 ± 0.30	4.20 ± 0.50
s*R*_g_ limits [from Guiner approximation]	0.50–1.34	0.60–1.30
*D*_max_ (nm)	10.10	12.70
oligomeric state	monomeric	homodimeric
pore volume estimate (nm^3^)	88	167
**Molecular Mass (kDa)**		
estimated (probability)	53 (80%)	82 (65%)
from sequence	51	74
ambiguity score	0.0 (3D reconstruction is potentially unique)	
**Software Used**		
primary data reduction	DAWN pipeline (Diamond Light Source UK)	
data processing	ScÅtter IV, ATSAS 4.1	
modeling	GASBOR, DAMAVER, SREFLEX	
calculation of model intensities	CRYSOL	
SASBDB code	SASDYV6	SASDYW6
***q* = 4πsin(θ)/λ**		

The low-resolution SAXS envelope for both proteins was generated using the GASBOR program,^18^ by running 10 iterations and averaging and filtering using the DAMVER program.^19^ The final model fitting to experimental data was performed using the CRYSOL program.^20^ Flexible refinement of the Fba1 model against the experimental SAXS profile was performed with SREFLEX,^21^ which uses normal-mode analysis to deform the initial atomic model along its lowest-frequency modes and improve the agreement with the scattering data. The refined model was selected on the basis of the best fit to the experimental curve (lowest χ^2^) while retaining acceptable stereochemistry.

The SAXS data and models have been deposited in the Small Angle Scattering Biological Data Bank^22^ (SASBDB) and are available under the accession codes SASDYV6 and SASDYW6 for Pk and Fba1, respectively.

## 3 Results and Discussion

### 3.1 The Experimentally Determined 3D Structures of Fba1 and Pk are Consistent with the *In* Silico-Predicted Models

IC is responsible for high mortality rates, accounting for approximately one million deaths annually.^23^ Interestingly, in recent years, *N. glabratus* has emerged as the leading cause of IC.^23^ The identification of proteins as potential therapeutic targets is of fundamental importance; these proteins must be involved in critical metabolic pathways or cellular functions whose inhibition or disruption results in the death of the pathogen. Furthermore, they must be structurally distinct between the host and the pathogen to subsequently design a selective drug against *Candida* that is relatively harmless to the patient.^24,25^ The enzymes fructose-1,6-bisphosphate aldolase (Fba1) and pyruvate kinase (Pk) are candidates that meet these criteria. Although the human host possesses these enzymes in its metabolic pathways, both Fba1 and Pk differ structurally from the enzymes of *N. glabratus*.^7^ In a mouse model, these two proteins have been shown to confer immune protection to mice against *C. albicans* and *N. glabratus*.^8^ In the case of Fba1, it is an enzyme considered a therapeutic target and vaccine candidate against a wide variety of pathogenic organisms because it has been shown to promote virulence by binding to plasminogen, aiding adhesion to host cells, as well as their immunomodulation.^26,27^ Candidemia has been detected in the serum of patients with the condition.^27^ Pk has been proposed as a therapeutic target primarily against parasitic and bacterial pathogens.^28–30^ Thus, Fba1 and Pk have been shown to play important roles in the metabolism and pathogenesis of various pathogenic microorganisms, including *Candida* species. Elucidating the 3D structure of both enzymes will help: (i) shed light on how drugs currently used in patients with CF or those with resistance may interact with and/or inhibit these enzymes; (ii) structurally modify drugs used against these pathogens; (iii) facilitate the design and synthesis of new antifungals against *Candida* species. The 3D structures of Fba1 and Pk from *N. glabratus* have not been elucidated; in this regard, our research group has been working for over a decade to obtain them using Small-Angle X-ray Scattering (SAXS), the technique that allowed us to obtain the corresponding structures, the oligomeric state, and assessing the 3D model predicted using AlphaFold3.^31^ The proteins obtained as described at Materials and Methods session using protocols previously described.^8,15^ To this end, size-exclusion chromatography coupled with SAXS (SEC–SAXS) was performed to assess the solution behavior and monodispersity of Pk and Fba1. The averaged scattering intensity plotted as a function of elution volume (Figure 1; SEC–SAXS was performed separately for Pk and Fba1 and the two profiles are overlaid only for comparison) shows well-defined elution peaks for both proteins, indicating the presence of discrete macromolecular species in solution.

**Figure 1:**
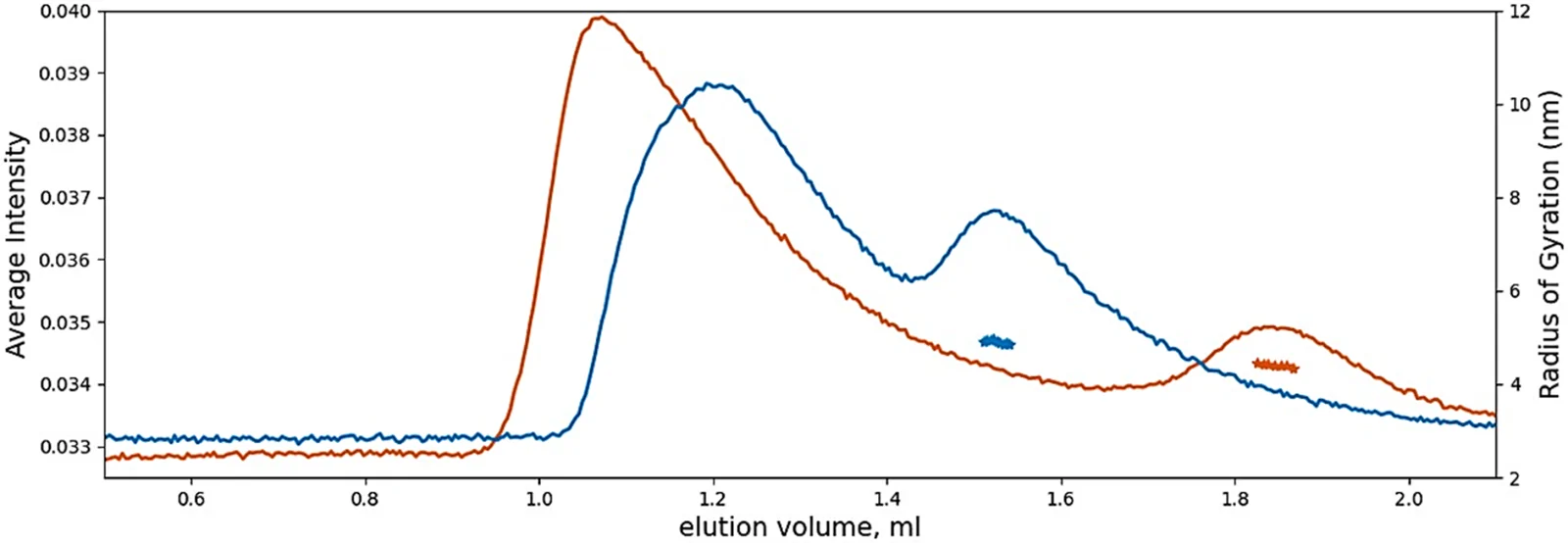
SEC–SAXS chromatogram showing the averaged SAXS scattering intensity as a function of elution volume for Pk (red) and Fba1 (blue). From the selected frames corresponding to the subtracted sample–buffer region across the elution peaks, the radius of gyration (*R*_g_) was estimated and is indicated by asterisks (*) in the corresponding colors. The *R*_g_ values remain consistent across the frames spanning the elution peak.

Pk elutes earlier (≈1.05–1.15 mL), consistent with a larger hydrodynamic size compared to Fba1, which elutes later (≈1.15–1.35 mL). For each elution peak, frames corresponding to the sample region were selected after buffer subtraction and analyzed independently. -radius-of-gyration (Rg) values estimated across the frames remain relatively constant throughout the central portion of the peaks (3.5 ± 0.6 nm and 4.4 ± 0.5 nm for Pk and Fba1, respectively), indicating structural stability and the absence of significant aggregation or interparticle interference during elution. Minor variations observed toward the edges of the peaks likely reflect dilution effects and partial overlap with the buffer baseline. The stability of *R*_g_ across the peak regions confirms that the selected frames correspond to homogeneous scattering species suitable for subsequent SAXS analysis and structural modeling. These frames were therefore averaged and used for further characterization and comparison with structural models. SAXS parameters are shown in Table 1, while Figure 2 summarizes the SAXS experimental data and their principal data analysis.

**Figure 2:**
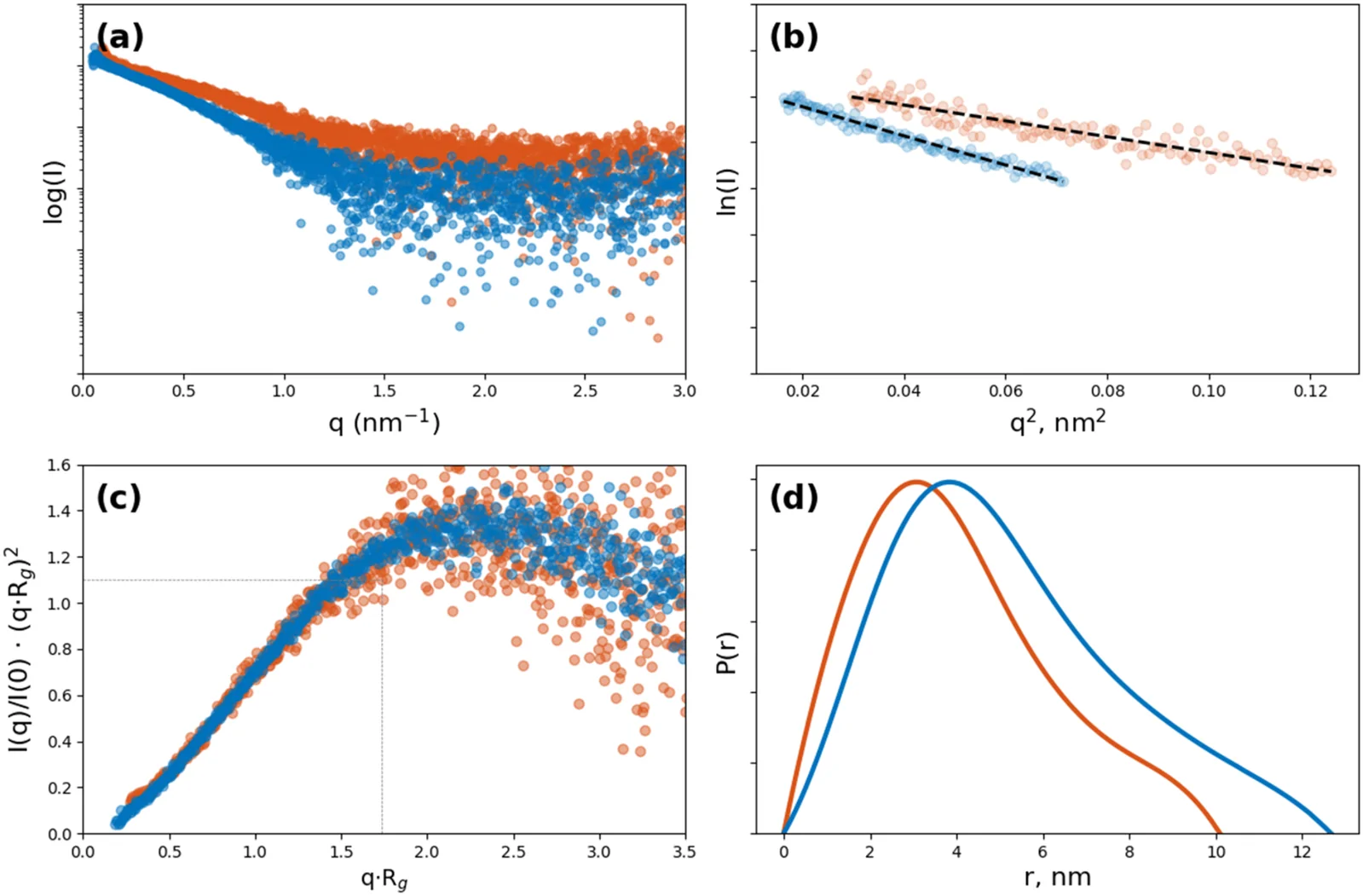
Pk (red) and Fba1 (blue). (a) Experimental SAXS; (b) Guiner region and the linear fit from which Rg is estimated; (c) Dimensionless Kratky plot. The intersection of the dotted black trace corresponds to the value of bovine serum albumin (a globular protein used as a standard in SAXS experiments); (d) Pair-distance distribution function (Pr) from which the maximum size (Dmax) was estimated.

The experimental scattering profiles (Figure 2a) for both proteins show smooth decays without upturns at low q, confirming the absence of aggregation. Guinier analysis^32^ (Figure 2b) shows linear behavior in the low-q region, supporting the reliability of the extracted Rg values and indicating good data quality. The radius of gyration (*R*_g_) obtained from the pair-distance distribution function P­(r) is 3.20 ± 0.3 nm for Pk and 4.21 ± 0.8 nm for Fba1, consistent with the Guinier analysis within the appropriate s*R*_g_ ranges, supporting the absence of significant interparticle effects. Both Pk and Fba1 exhibit bell-shaped dimensionless Kratky profiles characteristic of folded particles; both curves rise to a peak close to the reference position expected for a globular protein (intersection of the dashed lines), indicating that neither protein is unfolded. Fba1 shows a slightly more elevated profile at higher q*R*_g_, consistent with a modestly greater degree of extension or conformational flexibility than Pk. The pair-distance distribution functions P­(r) (Figure 2d) reveal distinct shapes for the two proteins. Pk displays a comparatively compact distribution (*D*_max_ ≈ 10 nm) with a moderate tail, consistent with an elongated, multidomain monomer, whereas Fba1 shows a broader distribution with a pronounced tail extending to larger distances (*D*_max_ ≈ 13 nm), indicative of a more extended architecture. Together with the radii of gyration (Pk, *R*_g_ ≈ 3.2 nm; Fba1, *R*_g_ ≈ 4.21 nm), these results indicate that Fba1 adopts a more extended conformation in solution than Pk, and support clear structural differences between the two proteins.

Molecular mass estimates derived via Bayesian inference of experimental SAXS data^17^ are in reasonable agreement with the theoretical values from sequence analysis, yielding ∼ 53 kDa (80% probability) for Pk versus an expected 51 kDa, and ∼ 82 kDa (65% probability) for Fba1 versus 74 kDa. These results support the assignment of the oligomeric states in solution as monomeric for Pk and homodimeric for Fba1. For Fba1 the homodimeric state was expected, whereas Pk was found to be monomeric rather than in the tetrameric state typical of most pyruvate kinases. Pyruvate kinases commonly exist in a concentration- and ligand-dependent monomer–dimer–tetramer equilibrium that shifts toward the tetramer at higher protein concentration. In our SEC–SAXS experiment, the sample was injected at 5 mg/mL and as is intrinsic to size-exclusion chromatography, diluted further on the column before reaching the X-ray beam; the effective concentration at the point of measurement is therefore substantially lower than the injection value. In addition, size-exclusion chromatography resolved an earlier aggregate peak from the peak corresponding to the species of interest, so that only the monodisperse, nonaggregated fraction was used for analysis. Under these dilute conditions and in the absence of activating ligands, the monomeric form is the expected predominant species, rather than necessarily the physiologically active oligomeric state. Structural data in solution obtained using SAXS for Fba1 allow for the evaluation of the conservation and divergence of its molecular dynamics under physiological conditions for future comparative evolution studies in yeasts. By contrasting global parameters such as the radius of gyration (*R*_g_), the maximum dimension (Dmax), and ab initio envelope models, it is possible to map how natural selection has shaped the flexibility of loop regions and the stability of their oligomeric states in fungal Class II. Meanwhile, structural data in solution obtained for the pyruvate kinase of *N. glabratus* provide a robust biophysical platform for further study of the comparative evolution of proteins within the ascomycete yeast clade. Unlike rigid crystallographic models, the characterization of the enzyme in solution reveals its dynamic landscape, allowing for the precise mapping of conformational fluctuations and the flexibility of the loops that protect the active site. From a phylogenetic perspective, evaluating these structural parameters in comparison with well-characterized homologues, such as the Pk of *Saccharomyces cerevisiae*, helps to decipher how small amino acid substitutions at the interfaces of allosteric domains have shaped the fine-tuning of glycolysis. These solution structures and oligomeric-state assignments provide a useful basis for future comparative studies of glycolytic enzymes across yeasts. Comparing the *N. glabratus* Fba1 and Pk with their counterparts in other fungi and in the human host could help identify species-specific structural features relevant to antifungal targeting and, in the longer term, inform questions about how the structure and regulation of these enzymes have diverged in fungi. The Porod volume estimates further corroborate differences in overall particle compactness between the two proteins. The ambiguity score of 0.0 indicates that the ab initio reconstructions are likely unique, increasing confidence in the derived low-resolution shapes (SASBDB^22^ entries: SASDYV6 and SASDYW6). This interpretation was further supported by a low-resolution Cα model generated using the GASBOR program^18^ through ten independent runs, followed by averaging and filtering with the DAMAVER program^19^ to obtain a consensus shape.

These low-resolution models aligned very well with the predicted models from AlphaFold3 (Figure 4). The model’s agreement with the experimental SAXS data was validated by fitting the calculated SAXS curve generated with CRYSOL^20^ to the experimental data (Figures 3 and 4).

**Figure 3:**
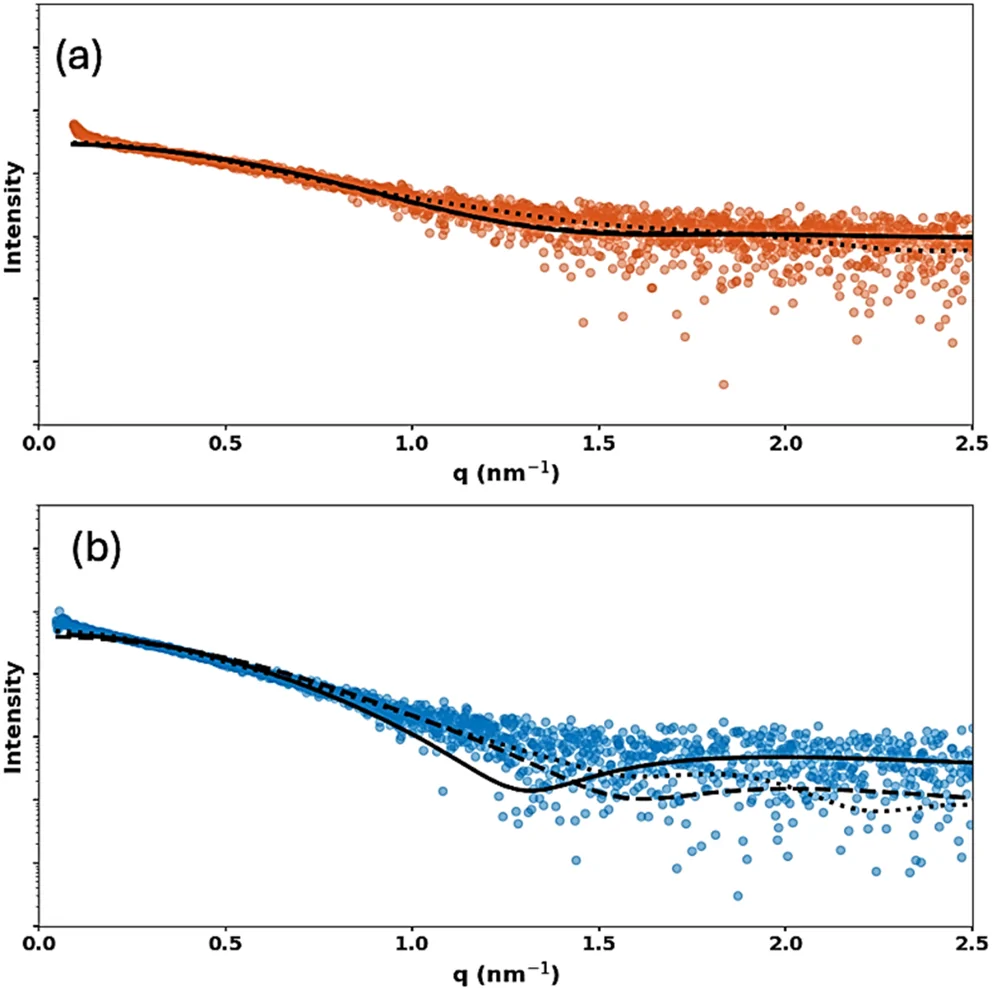
Pk (red, a) and Fba1 (blue, b). Comparison of the SAXS Cα model generated by GASBOR (black dashed line) with the AlphaFold3 prediction (black dotted line) and DM refinement (black dash-dotted line), fitted to the experimental scattering data.

**Figure 4:**
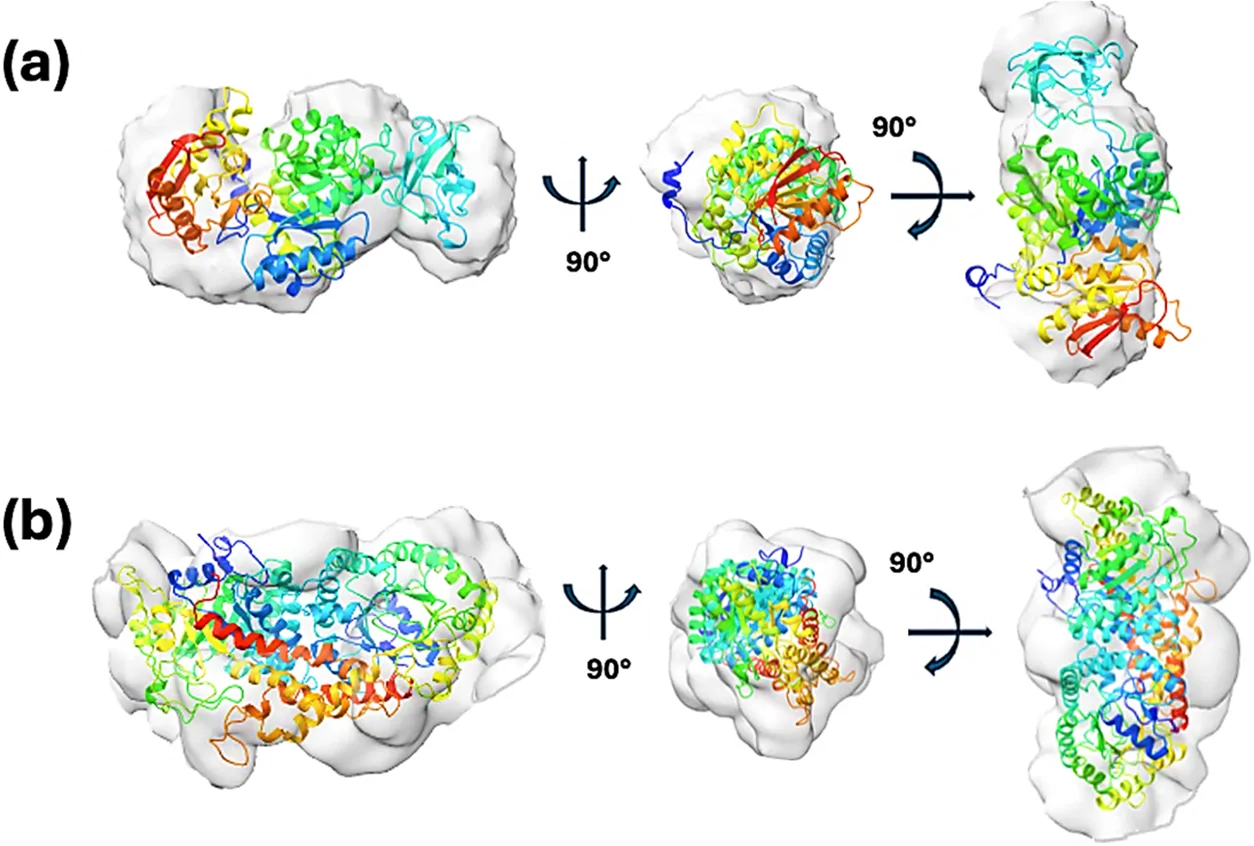
Structural comparison between the AlphaFold-predicted models and the SAXS ab initio envelopes obtained with GASBOR. (a) Pk and (b) **Fba1**. The AlphaFold models are shown as rainbow-colored cartoons (blue–red from N- to C-terminus), superimposed within the SAXS-derived Cα envelope reconstructed by GASBOR (gray surface). Three orthogonal projections, including a 90° rotation, are displayed to illustrate the agreement between the predicted atomic models and the low-resolution SAXS shapes.

In conclusion, the SAXS experiment was very useful not only for confirming the good condition of the protein in the buffer but also for demonstrating excellent agreement between the experimental data and the predictions.

### 3.2 Molecular dynamics for Fba1 and Pk

The MD simulations of Pk and Fba1 were started from the corresponding AlphaFold3 models, generated as a monomer and a homodimer respectively, in agreement with the oligomeric states indicated by SAXS. AlphaFold3 models are often more compact than proteins in solution, because the method is trained largely on crystal structures, where lattice packing favors tightly folded conformations and constrains flexible loops, termini, and linkers. Consistent with this, the SAXS-derived dimensions of both enzymes were larger than those of the initial AlphaFold3 models, as shown by the fit of the calculated scattering profiles for these models against the experimental data (Figure 3). The MD simulations were therefore aimed at observing how the structures evolve and whether this improves their agreement with the experimental SAXS data. The difference between the *R*_g_ of the initial models and that of the experimental data reflects both the difference in particle shape and the way each value is obtained. For all models (AlphaFold3 and MD), *R*_g_ is calculated with the same program, CRYSOL,^20^ directly from the atomic coordinates, which calculate to different value for it, one as shape *R*_g_ and another one as shell *R*_g_ (including a hydration shell), which is expected to be closer to that estimated from experimental. For the experimental data, in contrast, *R*_g_ is estimated from the Guinier approximation, the slope of the linearized low-q region (ln *I* versus *q* ^2^), over the range q*R*_g_ ≈ 0.6–1.3 and independently from the pair-distance distribution function P­(r) obtained with GNOM. The two estimates are in close agreement for both proteins (see Table 1), confirming that the experimental *R*_g_ values are robust and not artifacts of the fitting procedure. These values reveal two distinct behaviors. For Pk, the experimental *R*_g_ and Dmax are very closed with that of the AlphaFold3 model (*R*_g_ 3.20 nm and Dmax 8.6 nm; 10%), consistent with the mild overcompaction expected for AlphaFold predictions. The moderately skewed P­(r) reflects the elongated, multidomain architecture of the monomer rather than pronounced flexibility, in agreement with the uniformly confident Predicted Aligned Error (PAE) (Figure 5). For Fba1, the experimental *R*_g_ and Dmax are not far from that of the model (*R*_g_ 3.50 nm; ∼17% and Dmax 9.9; ∼20%); together with the pronounced skew of the P­(r) toward large r and the slightly elevated dimensionless Kratky profile (Figure 2c), this indicates an extended and conformationally flexible dimer in solution. Both proteins remain folded (their Kratky profiles rise to near the reference position for a globular particle), with Fba1 showing the greater extension. Importantly, the oligomeric-state assignments do not depend on *R*_g_ or overall shape but on the molecular weight, an independent observable. The MW values estimated from the experimental SAXS data (53 kDa for Pk and 82 kDa for Fba1) agree closely with the theoretical values calculated from the sequence for a monomer and a homodimer, respectively (51 and 74 kDa). As these estimates were obtained by a concentration-independent Bayesian consensus approach, the agreement provides direct support for the assignment of Pk as monomeric and Fba1 as homodimeric in solution, independently of their solution shape.

**Figure 5:**
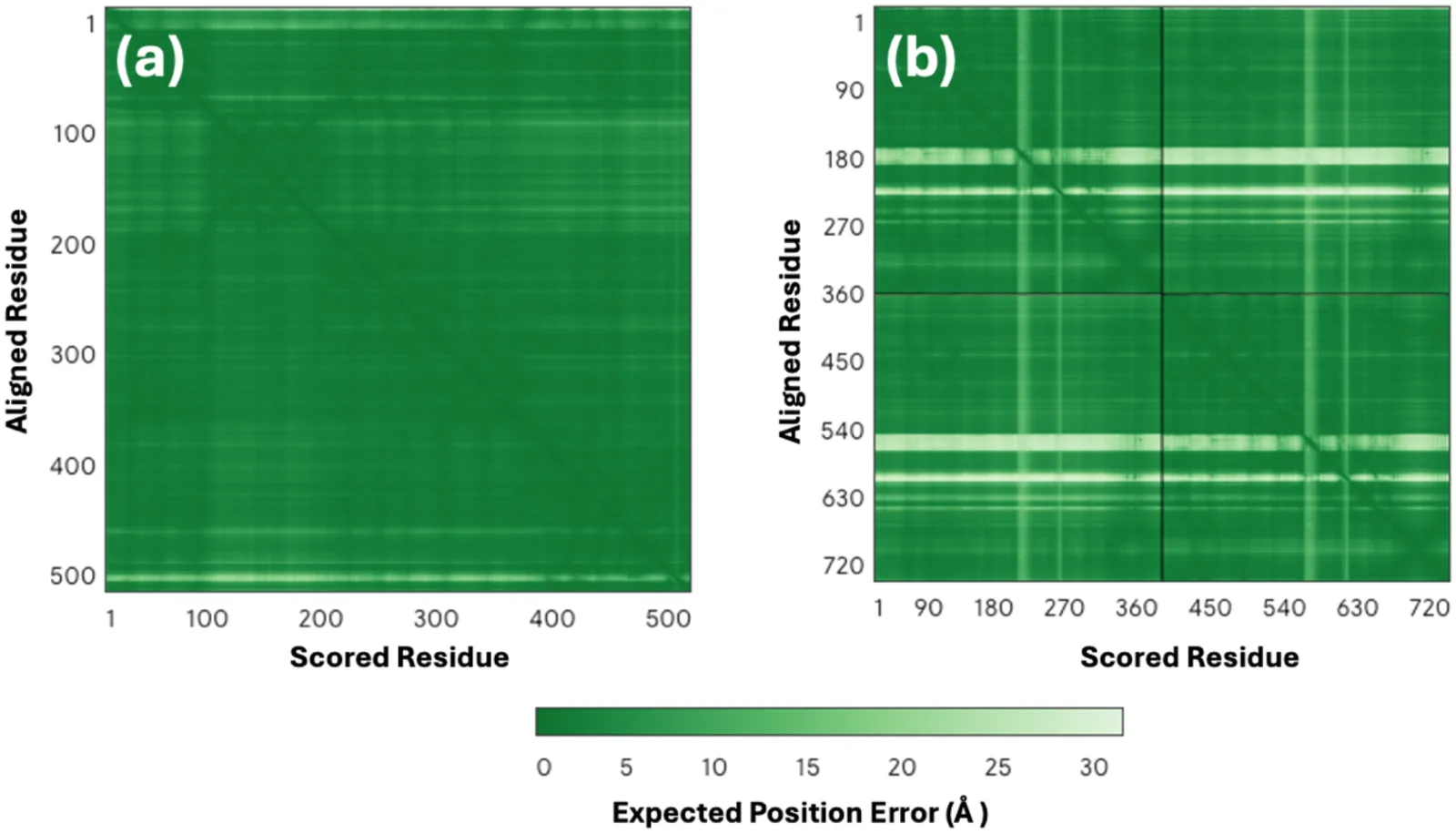
Predicted Aligned Error (PAE) of the AlphaFold3 models of Pk and Fba1. PAE matrices for (a) the Pk monomer and (b) the Fba1 homodimer. Each element (i, j) reports the expected positional error (in Å; color scale) of residue i when the predicted and true structures are aligned on residue j; darker colors indicate greater confidence in the relative positioning of the two residues. (a) For Pk, uniformly low PAE values across the entire ∼ 520-residue chain indicates a confidently predicted, well-ordered monomer, with only minor uncertainty at the termini. (b) For Fba1, the two low-error blocks along the diagonal correspond to the two protomers of the homodimer (chain border indicated by the black lines), and the confidently predicted off-diagonal quadrants indicate a well-defined dimer interface. The elevated PAE bands (lighter stripes) at residues ∼190–230 and ∼550–600 mark flexible loop and terminal regions, which coincide with the peaks of highest fluctuation in the MD RMSF analysis (Figure 6).

For MD simulations, the most probable ionization state of histidine at physiological pH was assigned during system preparation by Desmond module^33^ within Schrödinger suit (Schrödinger Release 2025–3: Desmond Molecular Dynamics System, D. E. Shaw Research, New York, NY, 2025. Maestro-Desmond Interoperability Tools, Schrödinger, New York, NY, 2025), and hydrogen atoms were placed based on the local environment. Each structure was solvated in a water box with at least 15 Å separating the protein atoms from the box edge, using SPC water. Na^+^ and Cl^–^ ions were added to neutralize the system and achieve a physiological concentration of 0.15 M. We used the OPLS4 ^34^ force field, which has been shown to provide reliable conformational stability—a factor crucial for studying proteins in defined structural states.^35,36^ A cutoff distance of 10 Å was applied for the van der Waals (vdW) and short-range interactions, while long-range electrostatic forces were calculated using the particle-mesh Ewald method.^37^ Subsequently, the systems were equilibrated at 300 K in the NVT ensemble using Nosé–Hoover chain thermostat,^38^ followed by equilibration at 300 K and 1 atm using Nosé–Hoover chain thermostat coupled to the Martyna–Tobias–Klein barostat.^39^ Finally, after removing the restraints on the heavy atoms, we performed 300 ns production runs for both systems under NPT conditions, with a trajectory frames saved every 100 ps. Root Mean Square Deviations (RMSD) and Root Mean Square Fluctuations (RMSF) were calculated using the trajectory analysis tools implemented in Desmond/Schrödinger suite (Figure 6).

**Figure 6:**
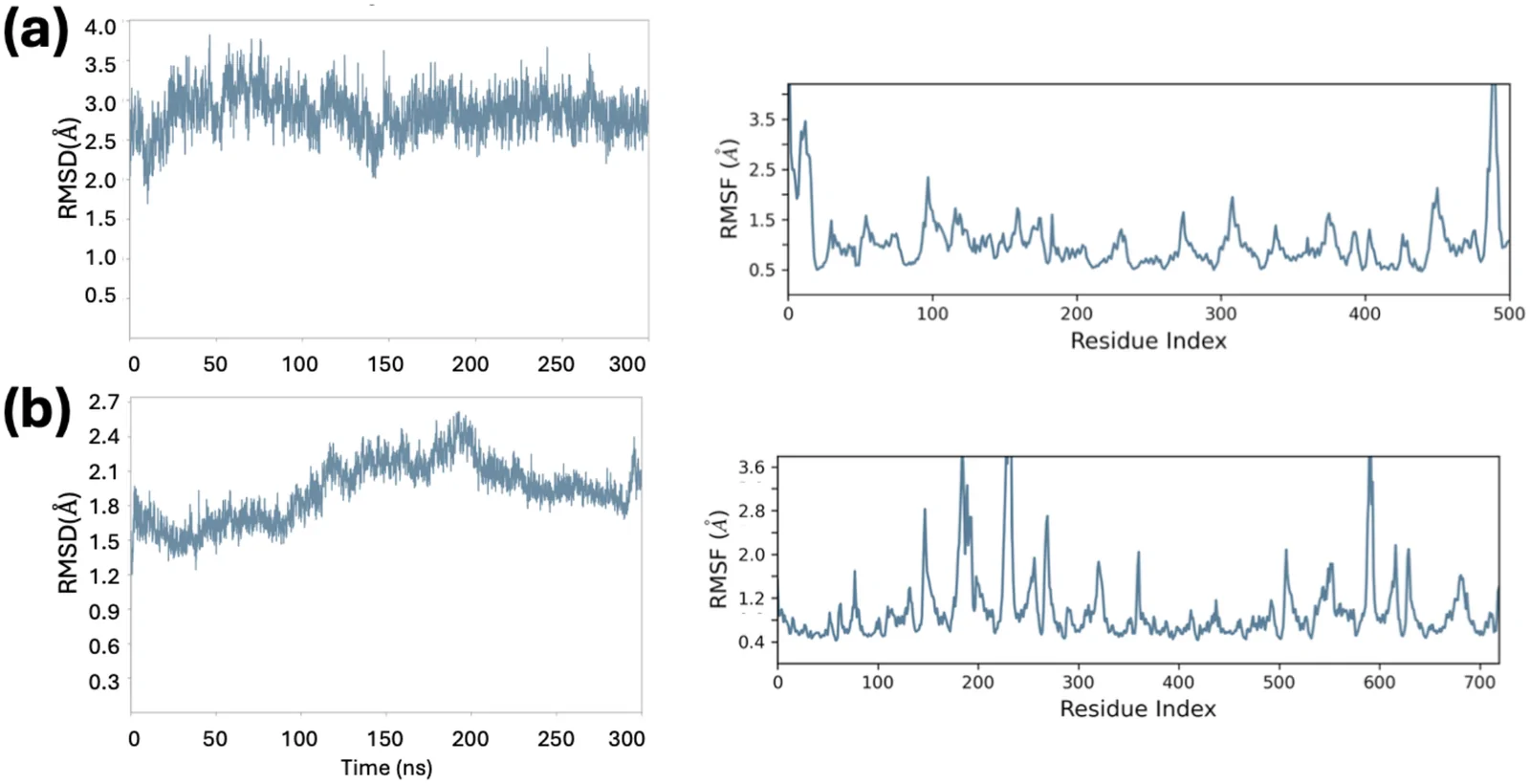
Molecular dynamics (MD) simulation starting from the AlphaFold model. (a) Pk. The time evolution of the backbone root-mean-square deviation (RMSD) relative to the AlphaFold starting structure shows an initial increase followed by stabilization, indicating relaxation toward a stable conformational ensemble. The root-mean-square fluctuation (RMSF) profile highlights a well-defined rigid core with increased flexibility at terminal regions and selected loop segments. The radius of gyration (*R*_g_) remains relatively stable after equilibration, with a slight increase compared to the initial model, consistent with a modest expansion in solution. (b) Fba1. RMSD analysis indicates structural stabilization of the dimer following an initial relaxation phase, with no evidence of large-scale unfolding. The RMSF profile reveals a predominantly rigid architecture, with localized flexibility in loop regions and at the termini. The *R*_g_ shows a more pronounced increase than PK, indicating a transition from a more compact AlphaFold-derived conformation to a slightly expanded, stable dimeric state in solution.

Specifically for PK, the RMSD rises rapidly from about 2.2–2.5 Å at the beginning to roughly ∼3.0 Å in the first part of the trajectory, then fluctuates mostly in the ∼2.6–3.1 Å range for the rest of the 300 ns, with occasional excursions toward ∼3.5–3.7 Å. There is no late monotonic drift, so the system appears to have reached a reasonably stable conformational ensemble after the initial relaxation from the AlphaFold starting model. That pattern is what we would expect when starting from an AlphaFold prediction: an early structural adjustment such as loops, termini, side-chain packing, and solvent-exposed regions— relax in explicit solvent, followed by sampling around a metastable state. In other words, the MD simulation does not suggest unfolding or major global instability, but it does indicate that the AlphaFold model likely requires moderate refinement before reaching a stable solution conformation.

The RMSF profile shows that most of the chain is relatively restrained, with many residues fluctuating around ∼0.6–1.2 Å, but there are clearly defined flexible regions. The strongest mobility is observed at the N-terminus and especially at the extreme C-terminus, where the RMSF rises above 4 Å. Additional moderate peaks are present around several internal loop regions, roughly near 90–120, 150–180, 300–320, 370–390, and 440–460. This suggests a protein with a stable folded core and mobile peripheral segments, particularly terminal regions and some loop insertions. For SAXS, these flexible segments can contribute disproportionately to scattering at low q, which may explain why a single rigid AlphaFold model often does not fit experimental data optimally.

The SSE analysis indicates that secondary structure is well maintained throughout the trajectory. The total SSE remains close to ∼ 52–55% over time with only small fluctuations, and the residue-vs-time map shows long-lived helical and strand segments rather than widespread conversion to loop/disordered states.

This is an important result: it means that the AlphaFold-based starting fold is globally consistent with a stable, compact architecture, while MD primarily refines relative positioning and flexible regions rather than disrupting the predicted fold (Figure 7).

**Figure 7:**
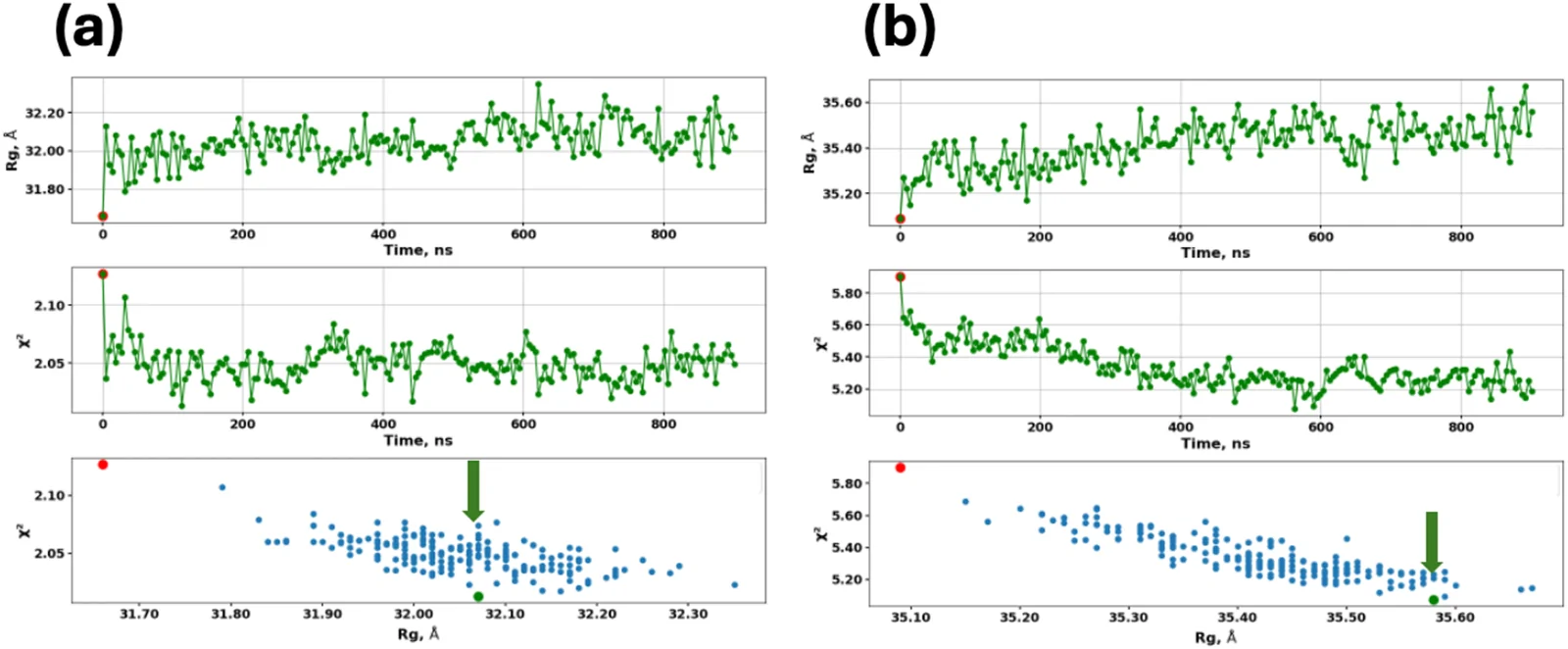
Molecular dynamics (MD) and SAXS-based analysis of Pk and Fba1 based on AlphaFold models. (a) Pk and (b) Fba1. For each protein: the backbone RMSD (relative to the starting structure) shows an initial relaxation followed by stabilization; the RMSF profile highlights a rigid core with increased flexibility at the termini and selected loops; the time evolution of the radius of gyration (*R*_g_, upper panel) and the corresponding agreement with the experimental SAXS data (χ^2^, middle panel) were evaluated every 15 frames; and the bottom panel shows the χ^2^–*R*_g_ relationship, with the green arrow indicating the frame of best agreement with the SAXS data. Pk undergoes only a modest expansion from the compact starting model, whereas Fba1 shows a more pronounced conformational adjustment toward its extended solution state; in both cases the frame selected for comparison with the experimental scattering is that of lowest χ^2^.

For Pk, the following conclusions are drawn: (i) the AlphaFold Pk model is broadly stable in MD; (ii) the system undergoes an initial relaxation of about ∼0.5–1 Å additional RMSD from the starting structure; (iii) the folded core remains preserved; (iv) termini and selected loops are flexible and likely dominate conformational heterogeneity.

We compare the simulation frames with SAXS experimental data tracking the evolution of *R*_g_ over time and assess agreement with them using the chi-square test (Figure 7). The initial AlphaFold structure has *R*_g_ = 32.0 Å. During MD, the values stabilize around *R*_g_ ≈ 31.8–32.0 Å, with fluctuations of roughly ± 0.15–0.2 Å (Figure 7). This indicates: (i) Rapid relaxation of the AlphaFold model; (ii) Systematic expansion of ∼0.8–1 Å; (iii) After relaxation, the ensemble samples a stable conformational basin; (iv) This is a very common behavior for AlphaFold models placed in explicit solvent MD, because AF predictions are usually slightly overcompact compared with solution ensembles measured by SAXS.^40,41^

Meanwhile, the lower plot shows that (i) Initial structure: χ^2^ ≈ 2.13; (ii) MD ensemble: χ^2^ ≈ 2.03–2.07; Thus, MD improves the agreement by roughly ∼5–7%. Additionally, there are important observations: (i) The improvement occurs immediately after the first relaxation. (ii) χ^2^ values fluctuate around ≈2.05, indicating that the ensemble samples similar SAXS-compatible conformations. (iii) No clear correlation between χ^2^ and large *R*_g_ shifts is visible, meaning SAXS differences probably arise from local domain motions or flexible loops, not global expansion. To better clarify we have shown in Figure 3 (black dashed-dotted line) the best model (indicated by a green arrow in Figure 7) fitting of the calculated SAXS curves respect to the experimental ones, where improvement cannot be distinguished, at least respect to the SAXS data. This found is consistent with SAXS theory, where scattering curves are sensitive to overall shape and domain arrangement rather than small fluctuations in compact proteins.

A χ^2^ around ∼2 indicates reasonable but not perfect agreement between the single best MD frame and the experimental data. This residual difference is consistent with the modest under-compaction of the AlphaFold3 model relative to the solution state and with limited local mobility of the termini and surface loops, rather than with large-scale conformational heterogeneity. Accordingly, for Pk a single representative conformation reproduces the overall size and shape reasonably well, and no ensemble description is required.

In the case of Fba1, the same MD–SAXS analysis indicates a clearer and larger relaxation than for Pk. The simulations were on a dimeric system (chains A and B, 360 residues each; 720 residues total) with 66,492 atoms. The average secondary-structure content is 42.77% helix, 12.41% strand, for a total SSE of 55.18%. The protein RMSD rises from about 1.5–1.7 Å initially to around 2.0–2.4 Å, with a broad maximum near ∼2.5–2.6 Å, then remains on a stable plateau, consistent with a preserved fold undergoing moderate conformational adjustment rather than unfolding. The RMSF profile shows a mostly rigid structure with pronounced flexible peaks in selected loop- -regions, especially around residues ∼170–230 and near ∼600, while the secondary-structure map indicates that the dimer remains strongly structured throughout the simulation.

From *R*_g_/χ^2^ plot, the initial AlphaFold-derived model starts at about *R*_g_ ≈ 35.0 Å and then relaxes to a trajectory ensemble centered roughly at *R*_g_ ≈ 35.4–35.6 Å with fluctuations of about ± 0.1–0.15 Å. Thus, compared to the initial model, MD results in a systematic expansion of approximately 0.6–0.8 Å.

At the same time, the SAXS agreement improves from approximately χ^2^ ≈ 5.9 for the initial model to approximately χ^2^ ≈ 5.2–5.4 for the MD-relaxed conformations, with the best frames near **∼**5.1–5.2. This means that, just as with PK, MD improves the agreement with SAXS, but for Fba1 the improvement is greater in absolute terms, because the initial fit is substantially worse. The trend is also more progressive: during the first part of the trajectory, as the structure becomes more expanded, the χ^2^ decreases steadily, and then both *R*_g_ and **χ** ^**2**^ fluctuate around a relatively stable region. This strongly suggests that the AlphaFold starting model is too compact for the solution state sampled by SAXS, and that the experimentally relevant conformation is a somewhat more open dimer. To better clarify, again, we have shown in Figure 3 (black dashed-dotted line) the best model (indicated by a green arrow in Figure 7) fitting of the calculated SAXS curves respect to the experimental ones (we note that selecting the single best-fitting frame does not substantially affect the interpretation, as SAXS is a low-resolution technique and is not sensitive to conformational fluctuations below ∼1 Å), where we can distinguish a large improvement, at least respect to the SAXS data. Compared with Pk, the interpretation is slightly different: (i) Pk already had a reasonably good initial agreement with SAXS, and MD only refined it modestly. (ii) Fba1 shows a stronger discrepancy between the initial model and the experimental scattering, so the MD relaxation appears more important in moving the structure toward the solution conformation. In both cases, the fold remains stable, but for Fba1 the SAXS data seem to require a more substantial adjustment in quaternary arrangement and/or interdomain packing.

The persistence of χ^2^ above 5, however, is significant. It indicates that, although MD improves the fit relative to the initial AlphaFold3 model, a single conformation remains insufficient to reproduce the experimental SAXS data. This residual mismatch is consistent with the pronounced extension of Fba1 in solution (*R*_g_ 42.1 Å, Dmax 12.7 nm) relative to the more compact model and may arise from several contributions: small variations in the relative arrangement of the two subunits, intersubunit breathing motions, and the mobility of the flexible surface loops and termini identified by both the PAE (Figure 5) and RMSF (Figure 6) analyses. Taken together, these features indicate that the solution scattering of Fba1 is better described by a conformational ensemble than by any single representative structure. To account for this residual discrepancy, the AlphaFold3 Fba1 dimer was subjected to normal-mode-based flexible refinement against the experimental SAXS data using SREFLEX.^21^ This approach deforms the initial model along its low-frequency normal modes to improve agreement with the scattering curve, and reduced χ^2^ from ≈5.9 for the starting model to ≈1.4, with the refined model reaching a shell *R*_g_ of 4.21 nm (42.1 Å), in close agreement with the experimental value (Figure 8). This indicates that a moderate, hinge-like opening of the compact AlphaFold3 dimer is sufficient to reproduce the extended solution conformation of Fba1.

**Figure 8:**
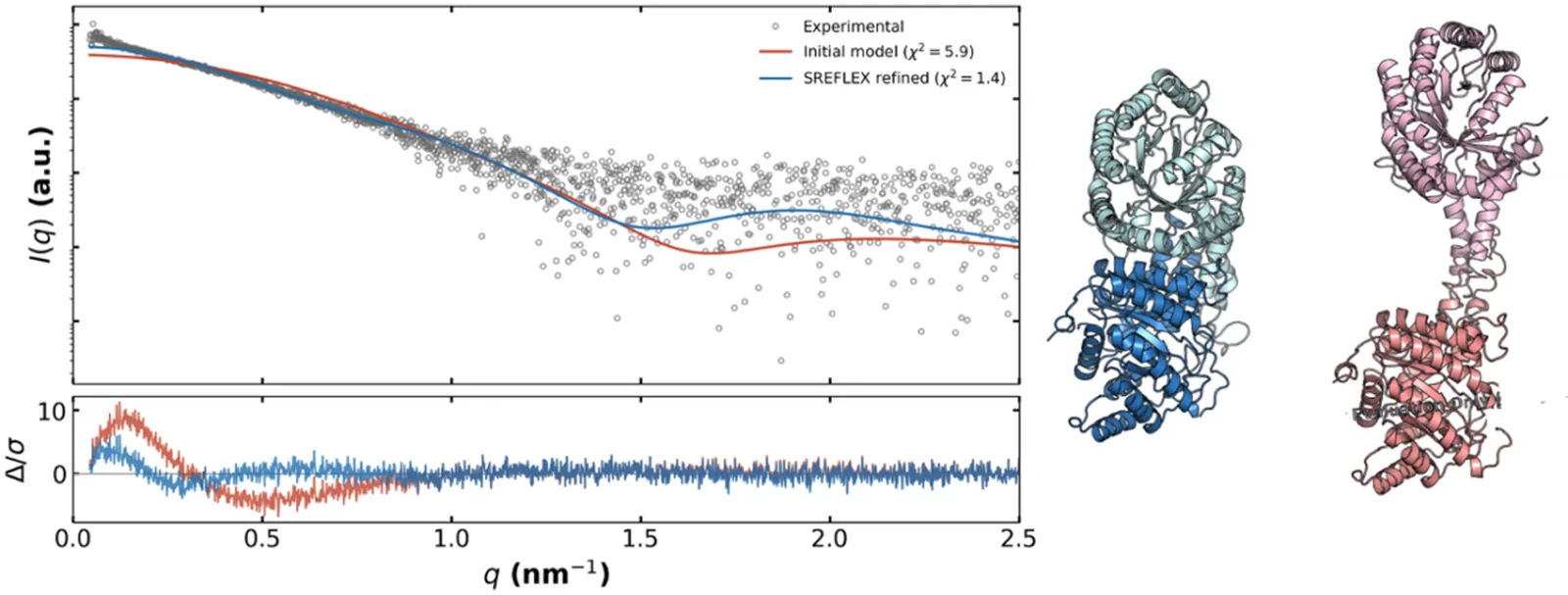
Flexible refinement of the Fba1 dimer against the experimental SAXS data (SREFLEX). (a) Experimental scattering profile (open circles) with the theoretical curves of the initial AlphaFold3 model (red; χ^2^ ≈ 5.9) and the SREFLEX-refined model (blue; χ^2^ ≈ 1.4); normalized residuals (Δ/σ) for both fits are shown below. (b) Cartoon representation of the initial compact model and the SREFLEX-refined, extended conformation, illustrating the hinge-like opening of the dimer. Flexible refinement along the low-frequency normal modes increases the shell *R*_g_ from 3.50 to 4.21 nm, matching the experimental value and reducing χ^2^.

The elucidation of the 3D structure of Fba1 and Pk from *N. glabratus* in this study opens the door to understanding these enzymes as potential therapeutic targets against this pathogen, and at the same time will allow us to understand the evolution of these proteins in yeasts. In this sense, both enzymes have been reported to be present in virtually all kingdoms of life.^42,43^ Specifically, in pathogenic microorganisms, it has been shown that Fba1 performs several nonglycolytic functions,^44^ data that are consistent with what was previously reported by our research group.^5–8^

## 4 Conclusions

This is the first study to describe the solution structure and oligomeric state of Fba1 and Pk from *N. glabratus*, combining SAXS with AlphaFold3 modeling and molecular dynamics. SAXS indicates that Pk is monomeric and Fba1 homodimeric in solution and provides low-resolution envelopes that are broadly consistent with the AlphaFold3 atomic models.

For both enzymes, the initial AlphaFold3 models are more compact than the solution data suggest, and molecular dynamics produced only a modest relaxation and expansion while preserving the folded core. For Fba1, the dimer remained structurally stable throughout the trajectory while undergoing a small, systematic expansion. For Pk, the monomeric model showed a comparable behavior, with the termini and flexible loops as the main mobile regions; the fit to the SAXS data improved only marginally and the absence of a clear correlation between χ^2^ and *R*_g_ indicates that the residual differences originate from local loop and domain motions rather than global expansion. For Fba1, by contrast, the initial AlphaFold3 model is substantially more compact than the solution data, and a single model does not reproduce the experimental scattering. Normal-mode-based flexible refinement of the model against the SAXS datas (SREFLEX) resolved this discrepancy, yielding an extended dimeric conformation in close agreement with the experimental value, indicating that a moderate, hinge-like opening of the compact predicted dimer is sufficient to account for its extended solution state.

These findings should be interpreted considering the intrinsic limitations of the methods. SAXS is a low-resolution technique that reports the overall size, shape, and oligomeric state of a particle in solution but not atomic-level detail, and its signal is an ensemble- and time-average over all conformations and species present, which requires careful sample monodispersity, addressed here through the SEC-SAXS separation of aggregated material. The AlphaFold3 models are computational predictions rather than experimental structures and, being trained largely on crystallographic data, tend to underestimate the conformational expansion and flexibility of proteins in solution; molecular dynamics only partially compensates for this and remains dependent on the force field, simulation time scale, and starting model. Within these limits, the convergence of experimental SAXS and computational modeling provides a consistent description of the solution behavior of Fba1 and Pk, while a full atomic-resolution characterization will require complementary high-resolution methods such as X-ray crystallography or cryo-EM.
